# Gender differences in higher-order aberrations and refractive error in Japanese school children: the Kyoto Childhood Refractive Error Study (KRES)

**DOI:** 10.1007/s10384-025-01272-6

**Published:** 2025-09-02

**Authors:** Yo Nakamura, Osamu Hieda, Yoshinori Nakai, Mitsuko Nakata, Chie Sotozono, Shigeru Kinoshita

**Affiliations:** 1 Department of Ophthalmology, Kyoto Prefectural University of Medicine, 465 Kajii-cho, Hirokoji-agaru, Kawaramachi-dori, Kamigyo-ku, Kyoto, 602-0841 Japan; 2 Department of Medical Welfare, Osaka University of Human Sciences, Osaka, Japan; 3 Department of Biostatistics, Kyoto Prefectural University of Medicine, Kyoto, Japan; 4 Department of Frontier Medical Science and Technology for Ophthalmology, Kyoto Prefectural University of Medicine, Kyoto, Japan

**Keywords:** School-aged children, Myopia, Higher-order aberrations (HOAs), Gender, Axial length

## Abstract

**Purpose:**

To investigate and analyze gender-related differences in myopia prevalence and factors associated with myopia progression in school-aged children.

**Study design:**

Observational study.

**Methods:**

This study involved 2,298 eyes (boys = 1194 eyes; girls=1104 eyes) of 1149 school children (597 boys; 552 girls) in two elementary/junior high schools in Kyoto Japan, examined from 2013 through 2022. Gender differences in all grades were evaluated in regard to subjective and objective refractive error (RE), axial length (AL), corneal keratometry, higher-order aberrations (HOAs), and a questionnaire regarding environmental factors of myopia progression.

**Results:**

In the girls in all grades, corneal keratometry was steeper and AL was shorter (*p*<0.05), coma-like and total aberration (in 6mm) corneal HOAs, coma-like, spherical-like, and total aberration (in 6mm) ocular HOAs were larger (*p*<0.05), in addition, only spherical aberration (in 4mm) corneal HOAs were smaller. In all grades, no gender-related differences were found in myopia prevalence (Grade 1: boys = 6.1%; girls = 6.5%, Grade 9: boys = 60.4%; girls = 65.4%) as well as RE. The questionnaire findings revealed that in all grades the girls spent more time reading and less time playing mobile-phone-app games (*p*<0.001).

**Conclusions:**

In Japanese school children, AL was shorter in the girls than in the boys, although, no gender-related differences were observed in myopia prevalence. The steeper cornea in girls might be associated with that discrepancy, and partially with gender differences of HOAs. Gender-specific differences of AL and HOAs should be considered in the analysis of myopia progression in school-aged children.

**Supplementary Information:**

The online version contains supplementary material available at 10.1007/s10384-025-01272-6.

## Introduction

In East Asia, the prevalence of myopia in the native population is very high [1, 2]. However, governmental intervention in both Taiwan and Singapore to control the progression of myopia in school-aged children is proving successful [3–5]. In Japan, data regarding uncorrected visual acuity (VA) (UCVA) in the annual school healthcare checkup has been the only statistical data on the prevalence of myopia in school children. We have been conducting the ‘Kyoto Childhood Refractive Error Study’ (KRES), a study on the RE of elementary and junior high school children in the Prefecture of Kyoto, Japan [7–9] since 2013, and in 2021, a national survey on refractive errors (RE) was initiated in Japan [6].

There are several reports on gender-based differences of myopic prevalence in the published literature. In the 1990s, it was reported that myopia was more common in adult males than females [10], and recently it has been reported that it is more common in girls [11–14] and, conversely that there is no difference in prevalence between boys and girls [15], so it is likely that not only genetic but also environmental factors are involved. To date, various environmental factors, such as education, near-vision work time, and outdoor activity time, have been pointed out as risk factors for the progression of myopia [16–20].

Since there are gender-related differences in infant growth curves, it is likely that there are also gender-related differences in eye development. Physical growth in school-aged children is closely related to growth hormones, yet after puberty, growth becomes more closely related to sex hormones. It is reported that girls, whose sex hormones are more likely to be secreted early, grow taller first [21]. Another report shows that school-aged children with earlier peak height velocities experienced peak axial length (AL) and spherical equivalent velocities earlier than children in whom puberty occured later [22], thus clearly illustrating the biological differences between males and females. Moreover, it is demonstrated in the United States, Europe, Australia, and China that AL is longer and corneal keratometry is flatter in males than in females [15, 23–26], and in many studies, the growth curves of AL have been evaluated in a gender-specific manner.

The purpose of this present study was to investigate and analyze gender-based differences in myopia prevalence in native Japanese school-aged children via the examination of RE, AL, corneal keratomery, and higher-order aberrations (HOAs), as well as via a questionnaire on environmental factors that may possibly impact the myopia progression.

### Participants and methods

The protocols of this observational study were approved by the Ethics Review Committee of Kyoto Prefectural University of Medicine, Kyoto, Japan (Approval No. RBMR-E-467-5). This study was conducted in accordance with the tenets set forth in the Declaration of Helsinki, and followed the guidelines of the Ethics Review Committee, an explanatory document including an option to not participate was presented to all children prior to their involvement in the study.

### Study population

The participants in this study were students attending two elementary/junior high schools; Kyoto-City Ryofu Gakuen (School A) and Fukuchiyama-City Yakuno Gakuen (School B), these two institutions of learning were selected as being typical public schools by the Boards of Education of Kyoto City and Kyoto Prefecture.

In School A, the study population in the first year (2013) consisted of 6- to 9-year-old students enrolled in elementary school Grades 1 through 3. Participants in the second year of the study were 6- to 10-year-old students enrolled in elementary school Grades 1 through 4. Participants in the third year of the study were 6- to 11-year-old students enrolled in elementary school Grades 1 through 5. Participants in the fourth year of the study were 6- to 12-year-old students enrolled in elementary school Grades 1 through 6. The number of grades was then gradually increased. From the seventh through ninth year of the study (i.e., from 2020 through 2022), the study population consisted of 6- to 15-year-old students enrolled in elementary school Grades 1 through 6 and junior high school Grades 7 through 9, respectively. In School B, the first year of the examinations was 2014, and all participants were enrolled in the same manner as described above for School A (Online Resource 1).

Since it was conducted as a school health check, students in the support class were also examined as much as possible.

### Ocular examinations

For the annual ocular examinations at both schools, the instruments used for the examination were brought to the school, and all examinations were performed by accredited ophthalmologists or nationally certified orthoptists.

In all participants, monocular distance UCVA and best-corrected VA (BCVA) examinations were performed, first in the right eye and then in the left eye with a Landolt ring at 5-meter distance. The findings were converted to Logarithm of the Minimum Angle of resolution (LogMAR) VA.

Objective RE and pupil distance were measured using the WR-5100K open-field binocular auto refractometer/keratometer (Grand Seiko) set to a 5-meter distance index, and subjective RE was examined by orthoptists. If distance UCVA was better than LogMAR 0, subjective RE was set to 0D. When there was a difference between objective and subjective RE in a single participant, objective RE was measured again for confirmation.

AL was measured with an IOL Master^®^ 500 Optical Biometer (Carl Zeiss Meditec AG). HOAs, corneal keratometry, and pupil diameter were measured using a KR-1W Corneal Wavefront Analyzer (Topcon Corporation). The ocular and corneal HOAs were analyzed at a pupil diameter of 4.0mm and 6.0mm, and the root-mean-square (RMS) value from the third- to sixth-order of Zernike coefficients were calculated. From those coefficients, corneal and ocular spherical aberrations, coma-like aberrations, spherical-like aberrations, and total aberrations were calculated.

### Questionnaire on environmental factors

Each year, the parents of the children were instructed to complete a questionnaire form on the following lifestyle factors; (1) time spent on outdoor activities, (2) time spent reading, (3) time spent playing mobile-phone-app games, and (4) time spent watching television, as well as a question regarding whether or not the parents were myopic (spectacles or contact lens wearers). The time for each activity was divided into four stages (< 1 hour, < 2 hours, < 3 hours, and ≥ 3 hours). Moreover, beginning in 2016 a question regarding height and weight was added to the questionnaire.

### Variable definition

Myopia was defined as a subjective spherical equivalent (SE) of less than or equal to -0.50 diopters (D), in addition to a UCVA of more than LogMAR 0 [27, 28]. High myopia was defined as a subjective SE of less than or equal to -6.00D.

### Statistics analysis

For the statistical analysis, UCVA, BCVA, subjective and objective SE, keratometry (D), AL, AL (mm)/corneal radius (mm) ratio (AL/CR ratio), pupil diameter, pupil distance, HOAs, and questionnaire were investigated. The following data were excluded from the statistics analysis as outliers; 0 mm of AL, more than 1.0 μ RMS value of HOAs analyzed at 4 mm, and 2.0 μ RMS value of HOAs analyzed at 6 mm. Data of both eyes were used for the statistical analysis. A linear mixed model or generalized linear mixed model with participant as a random effect and gender as a fixed effect was used to compare the differences in mean or difference in proportion, respectively, of each factor. To account for intra-subject correlation arising from the use of both eyes, robust standard errors (Huber-White sandwich estimator) were used to provide valid inference despite the non-independence of observations [29]. In addition to the usual estimates, standardized estimates were calculated that adjusted for differences in the number of students in each grade as a sensitivity analysis.

Regarding HOAs it might be affected by the corneal refractive power. Thus, the association between HOAs and keratometry were investigated via linear mixed models. Data with missing values were not included in the analysis (complete case analysis). In all analyses, a *P*-value of < 0.05 (two-sided) was considered statistically significant. All data analysis was performed using the SAS version 9.4 (SAS Institute Inc.).

## Results

In this study, a total of 2298 eyes (1194 male eyes and 1104 female eyes) of 1149 school children (597 boys and 552 girls) were examined (Online Resource 2). There were 17 students (0.7%, 8 boys and 9 girls) enrolled in the support class, and 4 of those 17 students were unable to undergo any of the examinations due to either developmental or behavioral issues.

### Prevalence of myopia

**Fig. 1:**
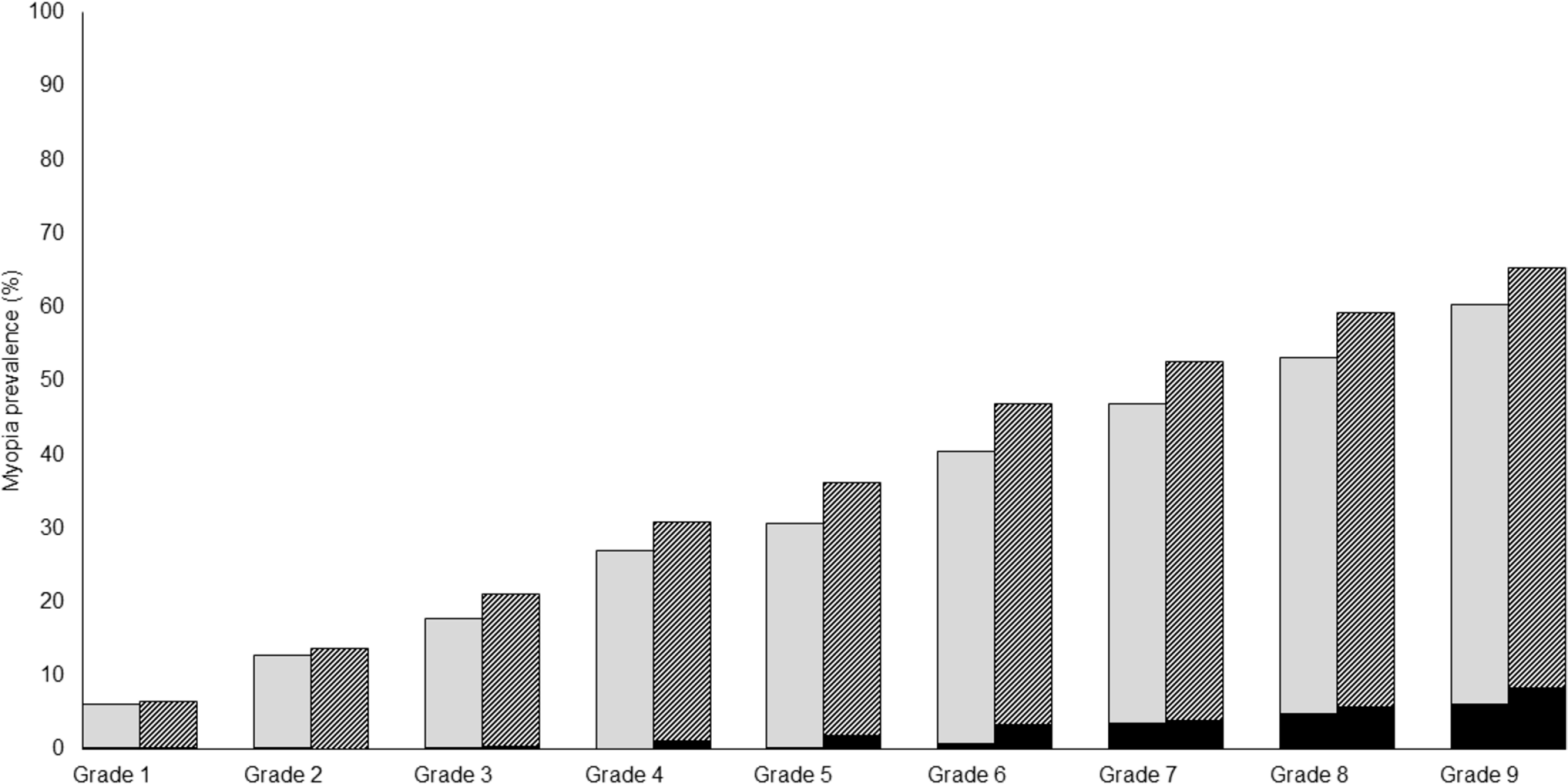
Myopia prevalence. Myopia was defined as a subjective spherical equivalent (SE) of less than or equal to -0.5 diopters (D), in addition to uncorrected visual acuity (UCVA) more than Logarithm of the Minimal Angle of Resolution (LogMAR) 0. High myopia was defined as a subjective SE of less than or equal to – 6.0D

The prevalence of myopia was 6.3% in Grade 1 (boys: 6.1%, girls: 6.5%), 43.5% in Grade 6 (boys: 40.5%, girls: 46.9%), and 62.7% in Grade 9 (boys: 60.4%, girls: 65.4%) (Fig. 1 and Online Resource 3). In each grade, no gender-associated difference in myopia prevalence was found. In all grades, the myopia prevalence was 26.8% for boys and 29.7% for girls, with no differences observed (*p* = 0.17). The proportion of high myopia was 2.0% in Grade 6 (boys: 0.8%, girls: 3.4%), and 7.2% in Grade 9 (boys: 6.1%, girls: 8.4%). However, statistical analysis could not be performed due to the small number of high myopia cases.

### VA and RE

In all grades, UCVA was significantly worse in girls than in boys (*p* = 0.004, Table 1). Although BCVA was also significantly worse in girls than boys in all grades, both were good LogMAR VA of < 0. Regarding the mean objective SE for all grades were – 0.60 ± 1.59D for boys and – 0.66 ± 1.78D for girls, with no significant difference observed between boys and girls (*p* = 0.81, the mean value in each grade is shown in Online Resource 4). Moreover, no significant difference in mean subjective SE was found between boys and girls in all grades (*p* = 0.33, boys: – 0.52 ± 1.46D, girls: – 0.61 ± 1.71D). The subjective astigmatism was significantly greater in girls than in boys (*p* = 0.003, boys: – 0.13 ± 0.38D, girls: – 0.20 ± 0.51D).

**Table 1:** Comparison of VA, RE, AL, keratometry, AL/CR ratio, pupil distance, pupil diameter, height, and weight between the boys and girls (all grades)

	Boys (all grades) *n* = 3364, 6728 eyes (mean ± SD)	Girls (all grades) *n* = 3145, 6290 eyes (mean ± SD)	*P*-value of fixed effect	*P*-value of fixed effect (standardized estimates) #
UCVA (logMAR)	0.159 ± 0.367	0.211 ± 0.405	0.004*	0.004*
BCVA (logMAR)	– 0.031 ± 0.087	– 0.024 ± 0.095	0.008*	0.01*
Objective Refraction (D)				
S	– 0.34 ± 1.55	– 0.38 ± 1.76	0.98	0.79
C	– 0.52 ±0.44	– 0.56 ± 0.50	0.06	0.09
SE	– 0.60 ± 1.59	– 0.66 ± 1.78	0.81	0.63
Subjective Refraction (D)				
S	– 0.46 ± 1.42	– 0.52 ± 1.70	0.56	0.45
C	– 0.13 ± 0.38	– 0.20 ± 0.51	0.003*	0.004*
SE	– 0.52 ± 1.46	– 0.61 ± 1.71	0.33	0.28
AL (mm)	23.73 ± 1.11	23.18 ± 1.07	< 0.001*	< 0.001*
Keratometry (D)				
Ks	43.5 ± 1.4	44.3 ± 1.5	< 0.001*	< 0.001*
Kf	42.6 ± 1.3	43.3 ± 1.4	< 0.001*	< 0.001*
AL/CR ratio				
Steep	3.06 ± 0.13	3.04 ± 0.14	0.01*	0.03*
Fat	3.00 ± 0.13	2.97 ± 0.13	< 0.001*	0.001*
Corneal astigmatism (D)	0.9 ± 0.5	1.0 ± 0.5	< 0.001*	< 0.001*
Pupil distance (mm)	57.4 ± 3.6	56.3 ± 3.4	< 0.001*	< 0.001*
Pupil diameter (mm)	6.23 ± 0.82	6.11 ± 0.87	0.01*	0.02*

	Boys (all grades) *n* = 1655	Girls (all grades) *n* = 1606	*P*-value of fixed effect	*P*-value of fixed effect (standardized estimates) #
Height (cm)	141.7 ± 16.4	139.9 ± 14.3	0.12	0.09
Weight (Kg)	37.0 ± 13.6	35.0 ± 11.3	0.04*	0.04*

*AL* axial length, *UCVA* uncorrected visual acuity, *BCVA* best-corrected visual acuity, *Ks* steep keratometry, *Kf* flat keratometry, *CR* corneal radius, *SE* spherical equivalent

^#^*P*-value: Standardized estimates adjusted for differences in the number of students in each grade as a sensitivity analysis were calculated. *P < 0.05

### AL, keratometry, AL/CR ratio, and pupil distance and diameter

In each grade and all grades, the AL was significantly shorter in girls than in boys (all grades: *p* < 0.001). The total mean AL was 23.73 ± 1.11 mm in boys and 23.18 ± 1.07 mm in girls (the mean value in each grade is shown in Online Resource 5). In each and all grades, the keratometry was significantly steeper in girls than in boys (all grades: *p* < 0.001). In the boys and girls, the total mean steep keratometry and total flat keratometry was 43.5 ± 1.4D and 44.3 ± 1.5D, and 42.6 ± 1.3D and 43.3 ± 1.4D, respectively. From Grade 1 through Grade 6, corneal astigmatism was significantly greater in girls than in boys, and a statistically significant difference was found for all grades (boys: 0.9 ± 0.5D, girls: 1.0 ± 0.5D) (*p* < 0.001, the mean value in each grade is shown in Online Resource 6). In all grades, the AL/CR ratio was significantly smaller in girls than in boys (steep AL/CR ratio: boys = 3.06, girls = 3.04 [*p* = 0.01]; flat AL/CR ratio: boys = 3.00, girls = 2.97 [*p* < 0.001]). In all grades, both pupil distance and pupil diameter were significantly smaller in girls than in boys (*p* < 0.001 and *p* = 0.01, respectively).

### HOAs

Regarding corneal HOAs, girls showed larger values than boys in all grades for 4 mm coma-like, 6 mm coma-like, and total aberrations. Only the corneal spherical aberration analyzed at a 4 mm diameter was smaller in girls than in boys in all grades. However, after adjusting for differences in the number of students in each grade, the difference in 4mm corneal coma-like aberrations was not significant (Table 2). Regarding ocular HOAs, coma-like, spherical-like, and total aberrations (at 6 mm analysis) were larger in girls than in boys in all grades (*p* < 0.05, the mean values in each grade are shown in Online Resources 7 through 10).

**Table 2:** Comparison of HOAs between boys and girls (all grades)

		Boys (all grades) *n* = 3364, 6728 eyes (mean ± SD)	Girls (all grades) *n* = 3145, 6290 eyes (mean ± SD)	*P*-value of fixed effect	*P*-value of fixed effect (standardized estimates)#
Cornea 4mm (μm)	Total	0.119 ± 0.059	0.121 ± 0.056	0.08	0.14
	Coma-like	0.103 ± 0.057	0.106 ± 0.054	0.03*	0.07
	Spherical	0.036 ± 0.024	0.033 ± 0.027	0.006*	0.01*
	Spherical-like	0.054 ± 0.029	0.053 ± 0.029	0.97	0.98
Cornea 6mm (μm)	Total	0.354 ± 0.134	0.368 ± 0.137	0.002*	0.004*
	Coma-like	0.275 ± 0.125	0.290 ± 0.125	< 0.001*	< 0.001*
	Spherical	0.178 ± 0.083	0.172 ± 0.096	0.07	0.14
	Spherical-like	0.209 ± 0.089	0.211 ± 0.098	0.84	0.71
Ocular 4mm (μm)	Total	0.110 ± 0.057	0.111 ± 0.056	0.58	0.56
	Coma-like	0.095 ± 0.055	0.096 ± 0.054	0.51	0.51
	Spherical	0.030 ± 0.028	0.026 ± 0.030	0.06	0.09
	Spherical-like	0.050 ± 0.028	0.050 ± 0.028	0.70	0.63
Ocular 6mm (μm)	Total	0.380 ± 0.194	0.403 ± 0.212	0.008*	0.007*
	Coma-like	0.302 ± 0.161	0.317 ± 0.163	0.02*	0.02*
	Spherical	0.137 ± 0.148	0.135 ± 0.173	0.78	0.87
	Spherical-like	0.213 ± 0.141	0.229 ± 0.167	0.02*	0.01*

*HOAs* higher-order aberrations

4 mm analysis; coma-like aberrations: S3, spherical-like aberrations: S4

6 mm analysis; coma-like aberrations: S3+S5, spherical-like aberrations: S4+S6, total aberrations: S3+S4+S5+S6

^#^*P*-value: Standardized estimates adjusted for differences in the number of students in each grade as a sensitivity analysis were calculated. *P < 0.05

### Relationship between 6 HOAs that had gender-related difference and corneal keratometry

Even after adjusting for corneal keratometry, there were gender differences in HOAs (Table 3). For corneal aberrations, there was a difference in two of the three aberrations (4 mm spherical and 6 mm coma aberrations) when adjusting for steep keratometry, and in all aberrations when adjusting for flat keratometry. For ocular aberrations, there were gender differences in two of the three aberrations (6 mm total and 6 mm coma) when adjusting for flat keratometry.

**Table 3:** Results of adjusted keratometry (Ks, Kf) for 6 HOAs that showed gender-related differences

		Estimates	95% CI	*P*-value of fixed effect		Estimates	95% CI	*P*-value of fixed effect
*Corneal*								
4mm Spherical	Gender	– 0.0043	– 0.0062, – 0.0024	< 0.001*	Gender	– 0.0043	– 0.0062, – 0.0025	< 0.001*
	Ks (1D)	0.0019	0.0008, 0.0031	0.001*	Kf (1D)	0.0024	0.0011, 0.0038	< 0.001*
6mm Total	Gender	0.0046	– 0.0051, 0.0143	0.35	Gender	0.0137	0.0038, 0.0236	0.007*
	Ks (1D)	0.0138	0.0094, 0.0181	< 0.001*	Kf (1D)	0.0032	– 0.0018, 0.0082	0.21
6mm Coma-like	Gender	0.0112	0.0009, 0.0214	0.03*	Gender	0.0214	0.0110, 0.0317	< 0.001*
	Ks (1D)	0.0094	0.0050, 0.0137	< 0.001*	Kf (1D)	– 0.0039	– 0.0089, 0.0010	0.12
*Ocular*								
6mm Total	Gender	0.0086	– 0.0082, 0.0254	0.31	Gender	0.0231	0.0058, 0.0403	0.009*
	Ks (1D)	0.0177	0.0117, 0.0238	< 0.001*	Kf (1D)	– 0.0004	– 0.0076, 0.0068	0.91
6mm Coma-like	Gender	0.0042	– 0.0086, 0.0169	0.52	Gender	0.0174	0.0043, 0.0304	0.009*
	Ks (1D)	0.0137	0.0092, 0.0181	< 0.001*	Kf (1D)	– 0.0034	– 0.0086, 0.0018	0.20
6mm Spherical-like	Gender	0.0070	– 0.0062, 0.0202	0.30	Gender	0.0119	– 0.0013, 0.0252	0.08
	Ks (1D)	0.0111	0.0062, 0.0159	< 0.001*	Kf (1D)	0.0059	0.0005, 0.0114	0.03*

*CI* confidence interval, *D* diopters, *Ks* steep keratometry, *Kf* flat keratometry

Estimated gender difference, reference value is boys. *P < 0.05

### Questionnaire on environmental factors

**Fig. 2:**
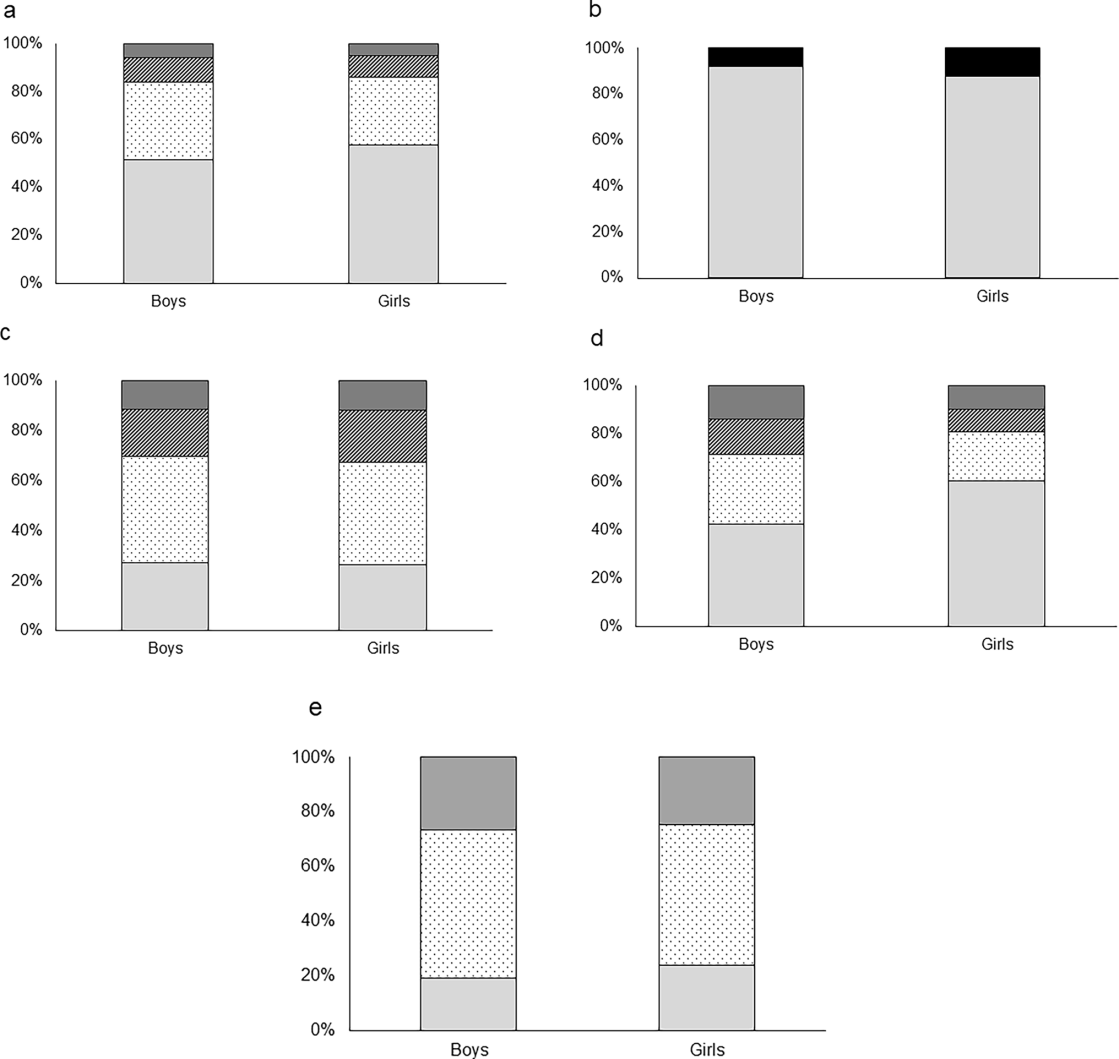
**a** Outdoor activity time (*p* = 0.06), **b** Reading time (*p* < 0.001), **c** Television time (*P* = 0.35), **d** Mobile-phone-app game time (*p* < 0.001), **e**. Numbers of subjects whose parents wear spectacles (*p* = 0.08). **a**, **c**, **d** <1 hour <2 hours <3 hours ≧3 hours. **b** <1 hour ≧1 hour. **e** 0 persons 1 person 2 persons

The questionnaire response rate was 89%. Among the factors in the questionnaire, significant differences between boys and girls were observed in reading time (girls > boys, *p* < 0.001) and mobile-phone-app game time in the all grades (boys > girls, *p* < 0.001) (Fig. 2 and Online Resource 11). As for trends by grade level, height was significantly lower in girls than in boys after Grade 6 (all *p* < 0.05), and weight was significantly lower in girls than in boys after Grade 8 (all *p* < 0.01, the mean value in each grade is shown in Online Resource 12). However, in all grades, no significant difference in height was found between boys and girls, yet weight was significantly lower in girls (*p* = 0.12 and *p* = 0.04, respectively, Table 1).

## Discussion

The findings in this observational study revealed that HOAs are larger in female than in male Japanese school children in Kyoto. That the corneal keratometry was steeper in the girls is thought to be one of the reasons. Moreover, the findings of steeper keratometry in the girls than in the boys is thought to be the reason why there was no difference in RE, even when the AL was short.

Several studies on the relationship between HOAs and myopia have been performed [30–34], however, and to the best of our knowledge, this is the first study to report the existence of gender-related differences. Our 6 mm ocular HOA values for school-aged children were 0.38 ± 0.20 μm for boys and 0.40 ± 0.21 μm for girls, close to the previously reported values of 0.37 ± 0.13μm by Kirwan and colleagues [31] and 0.35 ± 0.13 μm by Hiraoka and colleagues [33]. As shown in our findings, there was a difference between boys and girls in higher-order aberrations, even after adjusting for keratometry. It is suggested that differences in ellipsoid shape such as oblate spheroid or prolate spheroid, other than the corneal keratometry, are related. Our findings reveal differences in corneal and ocular aberrations between male and female school-aged children. Although statistically significant, the mean difference in higher-order aberrations between boys and girls was only 0.01 μm. This small difference is unlikely to affect visual function clinically, but may reduce retinal image clarity and be associated with axial elongation or myopia progression. Further investigation is needed to better elucidate the relationship between HOAs and myopia.

In all grades, girls had larger and steeper corneal refractive power and shorter AL than boys, thus indicating that girls have smaller eyes than boys. In this study, the pupil distance was smaller for girls than for boys in all grades, which is likely to be related to the gender-related differences in orbital cavity. The size of the orbital cavity grows by 75% from birth to 5 years of age, and from that point forward, it continues to grow until around 15 years of age and gender-related differences have been reported [35]. As the orbital cavity grows, the size of the eye also grows, and it is thought that there are gender differences in AL and corneal refractive power [15, 23–26]. However, it is reported that height and AL are correlated, so we thought it necessary to verify that point [22, 36, 37]. Our findings confirm that there is a difference in AL between boys and girls, even after adjusting for height (Online Resource 13). AL is usually used as an indicator of myopia progression, but it is necessary to take into account that there are gender differences in Japanese children as well.

There are also reports that incorporate the parameters of both AL and corneal refractive power and use the AL/CR ratio for the prediction of myopia. Our results show that girls had a smaller AL/CR ratio, which is inconsistent with the fact that there was no difference in myopia rate. It has been reported that the AL/CR ratio is smaller in girls [38, 39], and it is also reported that it does not match the myopia rate, which is consistent with our findings. Furthermore, a significant correlation was found when a scatter plot was analyzed between the AL/CR ratio and objective refraction. Our findings also reveal that the AL/CR ratio had a greater variability in girls, and that there were cases of severe myopia in girls in Grade 4 and above (Online Resource 14). However, the variability of the AL/CR ratio does require further investigation.

The myopia rate in this study was lower than that reported by Yotsukura and colleagues [40]. Hence, we considered the following two possible reasons for that difference. First, there is a possibility that there are differences in the myopia population depending on the geographical region, even within Japan [6]. And second, Yotsukura and colleagues determined myopia based on objective refraction measured by autorefractometer without cycloplegia, but we used UCVA and subjective refraction examined by nationally certified orthoptists. Furthermore, an open-field binocular auto-refractometer with an external fixation target we used is the method recommended by Senoo and colleagues [41].

In this study, there were no differences in myopia rate, subjective refraction, and objective refraction between the boys and girls. The girls spent significantly more time reading, and tended to spend less time playing outdoor than the boys. In addition, compared with the boys, the girls spent significantly less time playing mobile-phone-app games. Hence, the fact that environmental factors, overall, did not differ significantly between the boys and girls may help explain the fact that there was no significant difference in myopia prevalence.

The fact that girls had significantly greater subjective astigmatism than boys may be related to the fact that girls had lower uncorrected visual acuity in this study. Previous reports on gender differences in astigmatism indicate that girls have larger astigmatism than boys in Australia, India, and Nepal [42]. Changes in astigmatism and its relationship to myopia will be subjects for future study.

It should be noted that this study did have some limitations. First, the survey was conducted in one city (Kyoto), and the number of subjects in the survey was relatively small. Second, objective refraction was measured by non-cycloplegic refraction. However, we believe that we were able to obtain results similar to cycloplegic refraction by measuring refraction using the open-field autorefractometer with index placed far away.

In summary, the girls had a shorter AL and a steeper corneal keratometry compared to the boys. In addition, the girls had larger HOAs than did the boys, and that was somewhat related to steeper corneas. When evaluating AL and HOAs as factors related to the progression of myopia in school children, gender-specific evaluations should be performed.

## Supplementary Information

Below is the link to the electronic supplementary material.

Supplementary file1 (PDF 136 KB)

Supplementary file2 (PDF 102 KB)

Supplementary file3 (PDF 122 KB)

Supplementary file4 (PDF 188 KB)

Supplementary file5 (PDF 182 KB)

Supplementary file6 (PDF 180 KB)

Supplementary file7 (PDF 162 KB)

Supplementary file8 (PDF 161 KB)

Supplementary file9 (PDF 147 KB)

Supplementary file10 (PDF 161 KB)

Supplementary file11 (PDF 168 KB)

Supplementary file12 (PDF 148 KB)

Supplementary file13 (PDF 148 KB)

Supplementary file14 (PDF 258 KB)
