# Supplementary material for: Gender differences in higher-order aberrations and refractive error in Japanese school children: the Kyoto Childhood Refractive Error Study (KRES)
Source: Jpn J Ophthalmol. 2025 Sep 2;70(2):245–53. doi: 10.1007/s10384-025-01272-6 (PMC13091847; doi:10.1007/s10384-025-01272-6)
Supplement: Supplementary file 1 — Supplementary file1 (PDF 136 KB) [file 10384_2025_1272_MOESM1_ESM.pdf]

Online Resource 1 Number of participants (School A and B)

	Grade1	Grade2	Grade3	Grade4	Grade5	Grade6	Grade7	Grade8	Grade9
First year	n=112	n=115	n=103						
Second year	n=113	○	○	○					
Third year	n=131	○	○	○	○				
Fourth year	n=103	○	○	○	○	○			
Fifth year	n=86	○	○	○	○	○	○		
Sixth year	n=105	○	○	○	○	○	○	○	
Seventh year	n=99	○	○	○	○	○	○	○	○
Eighth year	n=92	○	○	○	○	○	○	○	○
Ninth year	n=90	○	○	○	○	○	○	○	○
