# Supplementary figures and images for: Gender differences in higher-order aberrations and refractive error in Japanese school children: the Kyoto Childhood Refractive Error Study (KRES)
Source: Jpn J Ophthalmol. 2025 Sep 2;70(2):245–53. doi: 10.1007/s10384-025-01272-6 (PMC13091847; doi:10.1007/s10384-025-01272-6)

**Online Resource 2** Number of cases in each grade

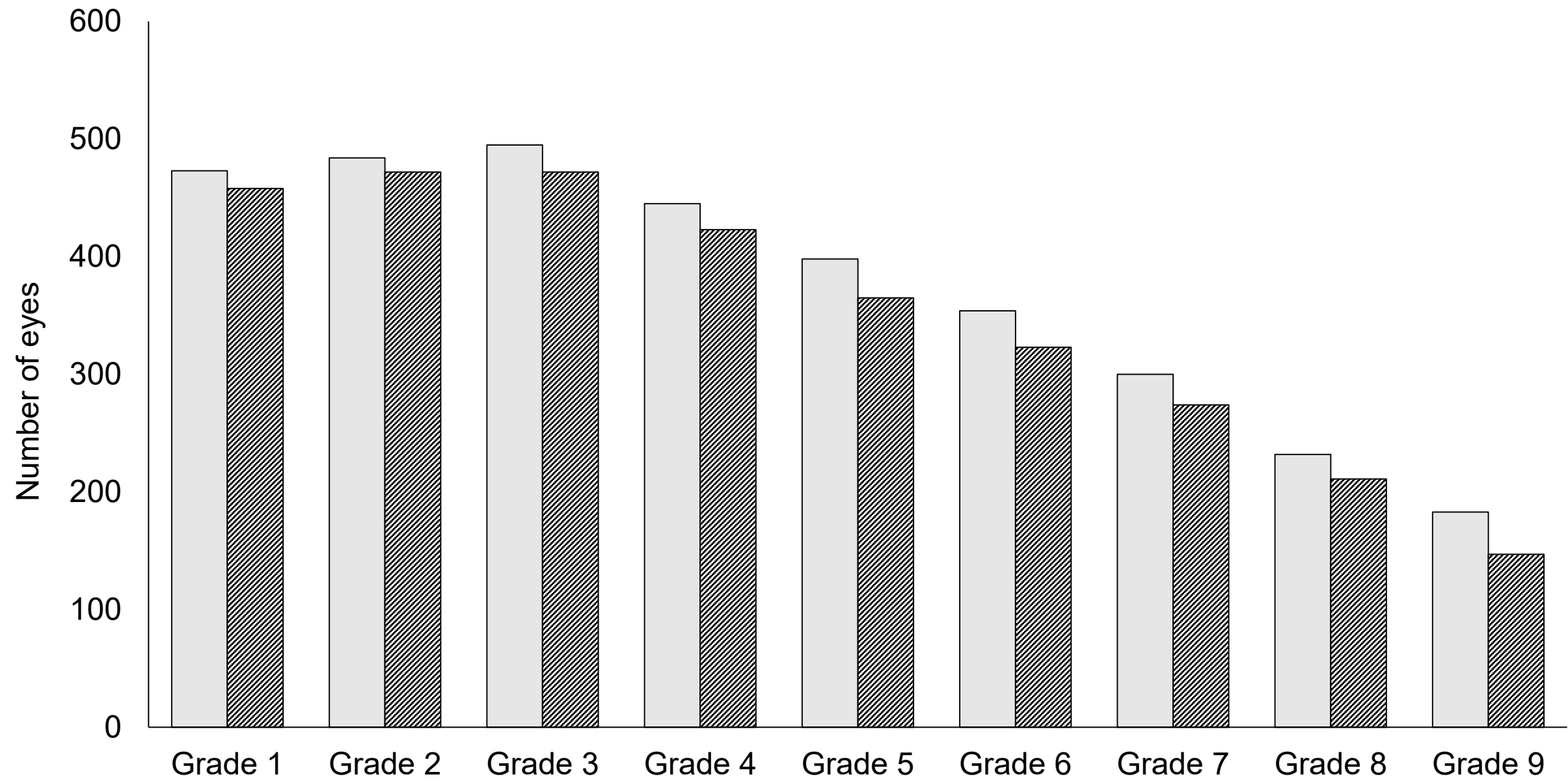

Supplement: Supplementary file 2 — Supplementary file2 (PDF 102 KB) [file 10384_2025_1272_MOESM2_ESM.pdf]
