# Supplementary material for: Gender differences in higher-order aberrations and refractive error in Japanese school children: the Kyoto Childhood Refractive Error Study (KRES)
Source: Jpn J Ophthalmol. 2025 Sep 2;70(2):245–53. doi: 10.1007/s10384-025-01272-6 (PMC13091847; doi:10.1007/s10384-025-01272-6)
Supplement: Supplementary file 3 — Supplementary file3 (PDF 122 KB) [file 10384_2025_1272_MOESM3_ESM.pdf]

Online Resource 3 Prevalence of myopia

		Grade 1	Grade 2	Grade 3	Grade 4	Grade 5	Grade 6	Grade 7	Grade 8	Grade 9
**Myopia prevalence**	**boys**	6.1 %	12.8 %	17.8%	26.9%	30.7%	40.5%	46.9%	53.1%	60.4%
	**girls**	6.5 %	13.7%	21.0%	30.9%	36.2%	46.9%	52.7%	59.2%	65.4%
**High myopia prevalence**	**boys**	0.2%	0.2%	0.2%	0.0%	0.3%	0.8%	3.6%	4.9%	6.1%
	**girls**	0.2%	0.1%	0.5%	1.3%	2.0%	3.4%	3.9%	5.8%	8.4%
