# Supplementary material for: Gender differences in higher-order aberrations and refractive error in Japanese school children: the Kyoto Childhood Refractive Error Study (KRES)
Source: Jpn J Ophthalmol. 2025 Sep 2;70(2):245–53. doi: 10.1007/s10384-025-01272-6 (PMC13091847; doi:10.1007/s10384-025-01272-6)
Supplement: Supplementary file 4 — Supplementary file4 (PDF 188 KB) [file 10384_2025_1272_MOESM4_ESM.pdf]

Online Resource 4 Comparison of subjective and objective refraction between boys and girls (each grade)

		Grade 1 (n=931)	p-value	Grade2 (n=956)	p-value	Grade3 (n=967)	p-value	Grade4 (n=868)	p-value	Grade5 (n=763)	p-value	Grade6 (n=677)	p-value	Grade7 (n=574)	p-value	Grade8 (n=443)	p-value	Grade9 (n=330)	p-value
**Objective refraction S (D)**	boys	0.37 ±0.85	0.04 *	0.22 ±0.97	0.69	-0.06 ±1.10	0.68	-0.24 ±1.33	036	-0.50 ±1.59	0.26	-0.78 ±1.71	0.20	-1.12 ±1.98	0.35	-1.37 ±2.11	0.29	-1.52 ±2.16	0.32
	girls	0.49 ±0.95		0.25 ±1.08		-0.10 ±1.32		-0.34 ±1.63		-0.65 ±1.86		-0.98 ±2.00		-1.31 ±2.17		-1.62 ±2.22		-1.81 ±2.34	
**Objective refraction C (D)**	boys	-0.50 ±0.38	0.02 *	-0.50 ±0.44	0.03 *	-0.50 ±0.43	0.32	-0.48 ±0.37	0.23	-0.51 ±0.43	0.41	-0.52 ±0.46	0.04 *	-0.59 ±0.50	0.86	-0.65 ±0.54	0.16	-0.58 ±0.54	0.60
	girls	-0.56 ±0.48		-0.57 ±0.49		-0.53 ±0.49		-0.52 ±0.47		-0.53 ±0.48		-0.62 ±0.67		-0.59 ±0.47		-0.58 ±0.47		-0.55 ±0.45	
**Objective refraction SE (D)**	boys	0.12 ±0.87	0.12	-0.03 ±0.99	0.96	-0.31 ±1.14	0.57	-0.48 ±1.34	0.29	-0.75 ±1.62	0.22	-1.04 ±1.76	0.13	-1.42 ±2.04	0.37	-1.69 ±2.18	0.38	-1.81 ±2.21	0.36
	girls	0.21 ±0.94		-0.04 ±1.06		-0.36 ±1.33		-0.59 ±1.62		-0.91 ±1.87		-1.29 ±2.08		-1.60 ±2.21		-1.91 ±2.25		-2.08 ±2.37	
**Subjective refraction S (D)**	boys	0.08 ±0.85	0.18	0.00 ±0.87	0.55	-0.16 ±0.96	0.69	-0.36 ±1.16	0.20	-0.54 ±1.42	0.15	-0.83 ±1.59	0.12	-1.14 ±1.86	0.39	-1.39 ±2.06	0.43	-1.51 ±2.07	0.37
	girls	0.16 ±0.91		0.04 ±1.08		-0.19 ±1.28		-0.49 ±1.6		-0.72 ±1.79		-1.06 ±1.93		-1.31 ±2.17		-1.58 ±2.30		-1.76 ±2.30	
**Subjective refraction C (D)**	boys	-0.11 ±0.34	0.09	-0.09 ±0.36	0.002 *	-0.09 ±0.37	0.006 *	-0.11 ±0.31	0.05	-0.15 ±0.35	0.04 *	-0.18 ±0.42	0.003 *	-0.20 ±0.47	0.48	-0.24 ±0.48	0.54	-0.21 ±0.44	0.74
	girls	-0.16 ±0.49		-0.18 ±0.54		-0.16 ±0.47		-0.17 ±0.51		-0.22 ±0.53		-0.30 ±0.59		-0.24 ±0.50		-0.21 ±0.43		-0.23 ±0.38	
**Subjective refraction SE (D)**	boys	0.03 ±0.82	0.34	-0.05 ±0.88	0.97	-0.21 ±0.99	0.40	-0.40 ±1.16	0.11	-0.61 ±1.46	0.10	-0.91 ±1.65	0.06	-1.24 ±1.95	0.38	-1.50 ±2.14	0.48	-1.62 ±2.14	0.39
	girls	0.08 ±0.88		-0.05 ±1.04		-0.27 ±1.27		-0.56 ±1.56		-0.82 ±1.80		-1.19 ±1.99		-1.41 ±2.22		-1.67 ±2.34		-1.86 ±2.36	

S, spherical; C, cylindrical; SE, spherical equivalent, mean ± SD   * P-value<0.05
