# Supplementary material for: Gender differences in higher-order aberrations and refractive error in Japanese school children: the Kyoto Childhood Refractive Error Study (KRES)
Source: Jpn J Ophthalmol. 2025 Sep 2;70(2):245–53. doi: 10.1007/s10384-025-01272-6 (PMC13091847; doi:10.1007/s10384-025-01272-6)
Supplement: Supplementary file 5 — Supplementary file5 (PDF 182 KB) [file 10384_2025_1272_MOESM5_ESM.pdf]

Online Resource 5 Comparison of AL, keratometry, AL/CR ratio between boys and girls (each grade)

		Grade 1 (n=931)	p-value	Grade 2 (n=956)	p-value	Grade 3 (n=967)	p-value	Grade 4 (n=868)	p-value	Grade 5 (n=763)	p-value	Grade 6 (n=677)	p-value	Grade 7 (n=574)	p-value	Grade 8 (n=443)	p-value	Grade 9 (n=330)	p-value
**AL (mm)**	boys	22.98 ±0.78	<0.001 *	23.25 ±0.83	<0.001 *	23.52 ±0.88	<0.001 *	23.73 ±0.97	<0.001 *	23.96 ±1.06	<0.001 *	24.19 ±1.13	<0.001 *	24.37 ±1.15	<0.001 *	24.57 ±1.23	0.001 *	24.63 ±1.28	0.01 *
	girls	22.40 ±0.70		22.68 ±0.76		22.96 ±0.83		23.21 ±0.94		23.44 ±0.97		23.69 ±1.08		23.88 ±1.12		24.06 ±1.12		24.24 ±1.09	
**Keratometry Steep (D)**	boys	43.6 ±1.4	<0.001 *	43.6 ±1.4	<0.001 *	43.5 ±1.4	<0.001 *	43.5 ±1.4	<0.001 *	43.4 ±1.4	<0.001 *	43.4 ±1.4	<0.001 *	43.5 ±1.4	<0.001 *	43.5 ±1.4	<0.001 *	43.4 ±1.3	<0.001 *
	girls	44.5 ±1.4		44.5 ±1.4		44.4 ±1.4		44.3 ±1.5		44.3 ±1.5		44.2 ±1.4		44.3 ±1.4		44.2 ±1.5		44.0 ±1.5	
**Keratometry Flat (D)**	boys	42.8 ±1.3	<0.001 *	42.7 ±1.3	<0.001 *	42.7 ±1.3	<0.001 *	42.7 ±1.3	<0.001 *	42.6 ±1.3	<0.001 *	42.5 ±1.3	<0.001 *	42.5 ±1.3	<0.001 *	42.5 ±1.3	<0.001 *	42.4 ±1.2	<0.001 *
	girls	43.5 ±1.4		43.5 ±1.3		43.4 ±1.3		43.4 ±1.4		43.3 ±1.4		43.2 ±1.3		43.2 ±1.3		43.2 ±1.3		43.0 ±1.3	
**AL/CR ratio steep**	boys	2.97 ±0.08	<0.001 *	3.00 ±0.09	0.008 *	3.03 ±0.10	0.03 *	3.05 ±0.11	0.60	3.08 ±0.12	0.93	3.11 ±0.13	0.71	3.14 ±0.15	0.27	3.16 ±0.15	0.38	3.17 ±0.15	0.86
	girls	2.95 ±0.08		2.99 ±0.09		3.02 ±0.10		3.05 ±0.12		3.08 ±0.13		3.10 ±0.14		3.13 ±0.15		3.15 ±0.16		3.16 ±0.16	
**AL/CR ratio flat**	boys	2.91 ±0.08	<0.001 *	2.95 ±0.09	<0.001 *	2.97 ±0.10	0.001 *	3.00 ±0.11	0.11	3.02 ±0.12	0.38	3.04 ±0.13	0.15	3.07 ±0.14	0.14	3.09 ±0.14	0.39	3.10 ±0.15	0.83
	girls	2.88 ±0.08		2.92 ±0.09		2.95 ±0.10		2.98 ±0.12		3.01 ±0.13		3.03 ±0.14		3.05 ±0.14		3.08 ±0.15		3.09 ±0.15	

AL, axial length; CR, corneal radius, mean ± SD   * P-value<0.05
