# Supplementary material for: Gender differences in higher-order aberrations and refractive error in Japanese school children: the Kyoto Childhood Refractive Error Study (KRES)
Source: Jpn J Ophthalmol. 2025 Sep 2;70(2):245–53. doi: 10.1007/s10384-025-01272-6 (PMC13091847; doi:10.1007/s10384-025-01272-6)
Supplement: Supplementary file 6 — Supplementary file6 (PDF 180 KB) [file 10384_2025_1272_MOESM6_ESM.pdf]

Online Resource 6 Comparison of visual acuity, corneal astigmatism, pupil distance and pupil diameter between boys and girls (each grade)

		Grade 1 (n=931)	p-value	Grade 2 (n=956)	p-value	Grade 3 (n=967)	p-value	Grade 4 (n=868)	p-value	Grade 5 (n=763)	p-value	Grade 6 (n=677)	p-value	Grade 7 (n=574)	p-value	Grade 8 (n=443)	p-value	Grade 9 (n=330)	p-value
**UCVA (LogMAR)**	boys	0.01 ±0.15	0.29	0.03 ±0.19	0.001 *	0.08 ±0.27	0.02 *	0.12 ±0.31	0.001 *	0.19 ±0.39	0.01 *	0.26 ±0.43	0.003 *	0.36 ±0.47	0.11	0.43 ±0.50	0.39	0.48 ±0.51	0.37
	girls	0.02 ±0.17		0.07 ±0.26		0.12 ±0.30		0.20 ±0.38		0.27 ±0.44		0.37 ±0.48		0.43 ±0.50		0.48 ±0.52		0.54 ±0.52	
**BCVA (LogMAR)**	boys	-0.03 ±0.07	0.05	-0.04 ±0.09	0.51	-0.03 ±0.10	0.65	-0.03 ±0.09	0.04 *	-0.03 ±0.09	0.49	-0.04 ±0.08	0.02 *	-0.02 ±0.06	0.66	-0.02 ±0.07	0.69	-0.02 ±0.13	0.81
	girls	-0.02 ±0.10		-0.03 ±0.11		-0.03 ±0.07		-0.02 ±0.07		-0.03 ±0.10		-0.02 ±0.10		-0.02 ±0.09		-0.02 ±0.10		-0.01 ±0.12	
**Corneal astigmatism (D)**	boys	0.9 ±0.5	<0.001 *	0.8 ±0.5	<0.001 *	0.8 ±0.50	<0.001 *	0.8 ±0.4	<0.001 *	0.8 ±0.5	<0.001 *	0.9 ±0.5	<0.001 *	1.0 ±0.6	0.09	1.0 ±0.6	0.84	1.0 ±0.6	0.79
	girls	1.0 ±0.5		1.0 ±0.6		1.0 ±0.5		1.0 ±0.6		1.0 ±0.6		1.1 ±0.6		1.1 ±0.6		1.0 ±0.5		1.0 ±0.5	
**Pupil distance (mm)**	boys	54.4 ±2.8	<0.001 *	55.4 ±2.7	<0.001 *	56.4 ±2.9	<0.001 *	57.2 ±2.9	<0.001 *	58.2 ±2.8	<0.001 *	59.0 ±2.9	0.002 *	60.1 ±3.1	<0.001 *	61.0 ±3.1	<0.001 *	61.9 ±2.9	<0.001 *
	girls	53.4 ±2.9		54.6 ±2.7		55.4 ±2.7		56.5 ±2.8		57.4 ±2.7		58.3 ±2.9		58.7 ±2.6		59.4 ±2.5		59.5 ±2.6	
**Pupil diameter (mm)**	boys	6.28 ±0.73	0.003 *	6.21 ±0.79	0.07	6.20 ±0.81	0.07	6.16 ±0.84	0.10	6.18 ±0.85	0.22	6.23 ±0.86	0.23	6.32 ±0.83	0.03 *	6.27 ±0.82	0.08	6.33 ±0.88	0.08
	girls	6.14 ±0.75		6.11 ±0.82		6.10 ±0.90		6.06 ±0.89		6.09 ±0.91		6.14 ±0.89		6.15 ±0.91		6.11 ±0.91		6.14 ±0.92	

BCVA, best-corrected visual acuity; LogMAR, Logarithm of the Minimum Angle of Resolution; UCVA, uncorrected visual acuity, mean ± SD ＊P-value<0.05
