# Supplementary material for: Gender differences in higher-order aberrations and refractive error in Japanese school children: the Kyoto Childhood Refractive Error Study (KRES)
Source: Jpn J Ophthalmol. 2025 Sep 2;70(2):245–53. doi: 10.1007/s10384-025-01272-6 (PMC13091847; doi:10.1007/s10384-025-01272-6)
Supplement: Supplementary file 7 — Supplementary file7 (PDF 162 KB) [file 10384_2025_1272_MOESM7_ESM.pdf]

Online Resource 7 Comparison of corneal HOAs analyzed at 4mm diameter between boys and girls (each grade)

		Grade 1 (n=931)	p-value	Grade 2 (n=956)	p-value	Grade 3 (n=967)	p-value	Grade 4 (n=868)	p-value	Grade 5 (n=763)	p-value	Grade 6 (n=677)	p-value	Grade 7 (n=574)	p-value	Grade 8 (n=443)	p-value	Grade 9 (n=330)	p-value
**Total**	boys	0.114 ±0.051	0.049 *	0.115 ±0.056	0.47	0.117 ±0.067	0.40	0.113 ±0.047	0.14	0.119 ±0.059	0.99	0.120 ±0.053	0.64	0.125 ±0.058	0.71	0.133 ±0.068	0.82	0.140 ±0.080	0.60
	girls	0.120 ±0.058		0.117 ±0.049		0.120 ±0.057		0.117 ±0.049		0.119 ±0.057		0.122 ±0.054		0.127 ±0.055		0.134 ±0.073		0.136 ±0.072	
**Coma-like**	boys	0.100 ±0.049	0.03 *	0.101 ±0.055	0.27	0.101 ±0.063	0.25	0.099 ±0.047	0.18	0.103 ±0.057	0.99	0.103 ±0.053	0.55	0.108 ±0.059	0.75	0.113 ±0.063	0.70	0.120 ±0.078	0.75
	girls	0.107 ±0.056		0.105 ±0.048		0.105 ±0.054		0.102 ±0.048		0.103 ±0.054		0.106 ±0.053		0.110 ±0.054		0.115 ±0.067		0.117 ±0.067	
**Spherical**	boys	0.030 ±0.023	0.39	0.032 ±0.026	0.14	0.035 ±0.024	0.002 *	0.035 ±0.021	0.01 *	0.037 ±0.026	0.30	0.039 ±0.022	0.09	0.041 ±0.021	0.86	0.047 ±0.024	0.09	0.044 ±0.029	0.27
	girls	0.029 ±0.023		0.029 ±0.021		0.030 ±0.032		0.031 ±0.026		0.035 ±0.028		0.036 ±0.026		0.040 ±0.023		0.042 ±0.028		0.040 ±0.037	
**Spherical-like**	boys	0.049 ±0.027	0.57	0.050 ±0.026	0.43	0.052 ±0.035	0.88	0.051 ±0.022	0.62	0.054 ±0.029	0.82	0.056 ±0.027	0.92	0.058 ±0.024	0.62	0.064 ±0.038	0.78	0.067 ±0.035	0.49
	girls	0.049 ±0.028		0.049 ±0.023		0.052 ±0.030		0.051 ±0.027		0.054 ±0.030		0.056 ±0.027		0.059 ±0.026		0.063 ±0.039		0.064 ±0.039	

HOAs, higher-order aberrations, mean ± SD μm *P-value<0.05
