# Supplementary material for: Gender differences in higher-order aberrations and refractive error in Japanese school children: the Kyoto Childhood Refractive Error Study (KRES)
Source: Jpn J Ophthalmol. 2025 Sep 2;70(2):245–53. doi: 10.1007/s10384-025-01272-6 (PMC13091847; doi:10.1007/s10384-025-01272-6)
Supplement: Supplementary file 8 — Supplementary file8 (PDF 161 KB) [file 10384_2025_1272_MOESM8_ESM.pdf]

Online Resource 8 Comparison of corneal HOAs analyzed at 6mm diameter between boys and girls (each grade)

		Grade 1 (n=931)	p-value	Grade 2 (n=956)	p-value	Grade 3 (n=967)	p-value	Grade 4 (n=868)	p-value	Grade 5 (n=763)	p-value	Grade 6 (n=677)	p-value	Grade 7 (n=574)	p-value	Grade 8 (n=443)	p-value	Grade 9 (n=330)	p-value
**Total**	boys	0.346 ±0.120	0.007 *	0.345 ±0.131	0.02 *	0.348 ±0.150	0.09	0.345 ±0.129	0.13	0.359 ±0.146	0.56	0.357 ±0.122	0.05	0.359 ±0.112	0.11	0.379 ±0.158	0.40	0.383 ±0.126	0.34
	girls	0.366 ±0.124		0.362 ±0.119		0.363 ±0.141		0.359 ±0.139		0.364 ±0.145		0.377 ±0.156		0.377 ±0.155		0.393 ±0.151		0.371 ±0.106	
**Coma-like**	boys	0.281 ±0.115	0.002 *	0.273 ±0.118	<0.001 *	0.274 ±0.141	0.09	0.270 ±0.122	0.14	0.276 ±0.129	0.59	0.273 ±0.120	0.06	0.269 ±0.109	0.15	0.283 ±0.144	0.42	0.287 ±0.121	0.54
	girls	0.302 ±0.118		0.297 ±0.118		0.288 ±0.120		0.281 ±0.128		0.280 ±0.125		0.291 ±0.138		0.284 ±0.135		0.295 ±0.132		0.279 ±0.106	
**Spherical**	boys	0.155 ±0.078	0.19	0.166 ±0.088	0.06	0.173 ±0.083	0.16	0.172 ±0.086	0.47	0.178 ±0.098	0.94	0.191 ±0.071	0.43	0.201 ±0.059	0.79	0.207 ±0.064	0.79	0.212 ±0.080	0.23
	girls	0.148 ±0.095		0.155 ±0.081		0.165 ±0.110		0.169 ±0.099		0.179 ±0.095		0.186 ±0.104		0.203 ±0.076		0.209 ±0.080		0.202 ±0.070	
**Spherical-like**	boys	0.191 ±0.075	0.89	0.196 ±0.097	0.64	0.202 ±0.088	0.59	0.204 ±0.082	0.67	0.214 ±0.106	0.59	0.220 ±0.077	0.33	0.227 ±0.073	0.16	0.242 ±0.096	0.59	0.243 ±0.081	0.24
	girls	0.190 ±0.086		0.194 ±0.075		0.206 ±0.108		0.208 ±0.096		0.218 ±0.108		0.226 ±0.108		0.238 ±0.102		0.248 ±0.107		0.233 ±0.070	

HOAs, higher-order aberrations, mean ± SD μm   * P-value<0.05
