# Supplementary material for: Gender differences in higher-order aberrations and refractive error in Japanese school children: the Kyoto Childhood Refractive Error Study (KRES)
Source: Jpn J Ophthalmol. 2025 Sep 2;70(2):245–53. doi: 10.1007/s10384-025-01272-6 (PMC13091847; doi:10.1007/s10384-025-01272-6)
Supplement: Supplementary file 9 — Supplementary file9 (PDF 147 KB) [file 10384_2025_1272_MOESM9_ESM.pdf]

Online Resource 9 Comparison of ocular HOAs analyzed at 4mm diameter between boys and girls (each grade)

		**Grade 1 (n=931)**	**p-value**	**Grade 2 (n=956)**	**p-value**	**Grade 3 (n=967)**	**p-value**	**Grade 4 (n=868)**	**p-value**	**Grade 5 (n=763)**	**p-value**	**Grade 6 (n=677)**	**p-value**	**Grade 7 (n=574)**	**p-value**	**Grade 8 (n=443)**	**p-value**	**Grade 9 (n=330)**	**p-value**
**Total**	boys	0.099 ±0.045	0.20	0.104 ±0.055	0.35	0.101 ±0.058	0.16	0.105 ±0.053	0.45	0.112 ±0.059	0.85	0.120 ±0.058	0.36	0.119 ±0.058	0.33	0.128 ±0.064	0.87	0.138 ±0.070	0.86
	girls	0.103 ±0.055		0.101 ±0.044		0.106 ±0.059		0.107 ±0.046		0.111 ±0.052		0.116 ±0.056		0.125 ±0.067		0.129 ±0.061		0.136 ±0.073	
**Coma-like**	boys	0.086 ±0.044	0.26	0.090 ±0.054	0.45	0.087 ±0.055	0.17	0.091 ±0.051	0.61	0.096 ±0.055	0.94	0.104 ±0.055	0.46	0.103 ±0.058	0.27	0.110 ±0.061	0.62	0.120 ±0.070	0.96
	girls	0.089 ±0.054		0.088 ±0.044		0.091 ±0.055		0.093 ±0.045		0.096 ±0.050		0.100 ±0.055		0.109 ±0.063		0.113 ±0.059		0.120 ±0.070	
**Spherical**	boys	0.021 ±0.026	0.40	0.024 ±0.026	0.14	0.027 ±0.028	0.07	0.029 ±0.026	0.33	0.032 ±0.029	0.34	0.035 ±0.028	0.06	0.038 ±0.026	0.13	0.041 ±0.027	0.05	0.039 ±0.029	0.41
	girls	0.019 ±0.028		0.021 ±0.027		0.024 ±0.030		0.027 ±0.028		0.030 ±0.030		0.031 ±0.029		0.034 ±0.032		0.034 ±0.034		0.036 ±0.032	
**Spherical-like**	boys	0.045 ±0.022	0.24	0.046 ±0.024	0.73	0.046 ±0.028	0.21	0.047 ±0.026	0.24	0.052 ±0.033	0.77	0.055 ±0.034	0.44	0.054 ±0.025	0.90	0.059 ±0.032	0.37	0.060 ±0.028	0.80
	girls	0.046 ±0.024		0.046 ±0.021		0.049 ±0.032		0.049 ±0.025		0.051 ±0.028		0.053 ±0.027		0.055 ±0.033		0.057 ±0.031		0.059 ±0.033	

HOAs, higher-order aberrations, mean ± SD μm
