# Supplementary material for: Gender differences in higher-order aberrations and refractive error in Japanese school children: the Kyoto Childhood Refractive Error Study (KRES)
Source: Jpn J Ophthalmol. 2025 Sep 2;70(2):245–53. doi: 10.1007/s10384-025-01272-6 (PMC13091847; doi:10.1007/s10384-025-01272-6)
Supplement: Supplementary file 10 — Supplementary file10 (PDF 161 KB) [file 10384_2025_1272_MOESM10_ESM.pdf]

Online Resource 10 Comparison of ocular HOAs analyzed at 6mm diameter between boys and girls (each grade)

		Grade 1 (n=931)	p-value	Grade 2 (n=956)	p-value	Grade 3 (n=967)	p-value	Grade 4 (n=868)	p-value	Grade 5 (n=763)	p-value	Grade 6 (n=677)	p-value	Grade 7 (n=574)	p-value	Grade 8 (n=443)	p-value	Grade 9 (n=330)	p-value
**Total**	boys	0.345 ±0.158	0.04 *	0.361 ±0.209	0.40	0.356 ±0.194	0.10	0.359 ±0.175	<0.001 *	0.381 ±0.194	0.07	0.407 ±0.190	0.47	0.424 ±0.198	0.04 *	0.450 ±0.211	0.20	0.471 ±0.208	0.54
	girls	0.366 ±0.186		0.371 ±0.203		0.375 ±0.190		0.406 ±0.230		0.407 ±0.216		0.418 ±0.207		0.464 ±0.238		0.480 ±0.221		0.491 ±0.226	
**Coma-like**	boys	0.279 ±0.138	0.06	0.293 ±0.172	0.94	0.282 ±0.158	0.10	0.282 ±0.145	0.001 *	0.298 ±0.156	0.15	0.321 ±0.159	0.83	0.332 ±0.165	0.04 *	0.355 ±0.181	0.19	0.372 ±0.183	0.48
	girls	0.295 ±0.144		0.293 ±0.155		0.297 ±0.149		0.315 ±0.161		0.314 ±0.167		0.323 ±0.158		0.363 ±0.184		0.380 ±0.184		0.390 ±0.193	
**Spherical**	boys	0.080 ±0.138	0.53	0.103 ±0.144	0.89	0.123 ±0.148	0.47	0.142 ±0.138	0.28	0.160 ±0.138	0.51	0.167 ±0.145	1.00	0.179 ±0.149	0.82	0.187 ±0.144	0.82	0.204 ±0.163	0.24
	girls	0.073 ±0.162		0.103 ±0.173		0.116 ±0.155		0.156 ±0.178		0.166 ±0.165		0.167 ±0.174		0.183 ±0.189		0.186 ±0.164		0.183 ±0.168	
**Spherical-like**	boys	0.188 ±0.109	0.11	0.196 ±0.143	0.14	0.201 ±0.141	0.22	0.204 ±0.132	0.007 *	0.220 ±0.145	0.06	0.229 ±0.145	0.21	0.242 ±0.151	0.11	0.253 ±0.156	0.37	0.264 ±0.154	0.76
	girls	0.200 ±0.144		0.209 ±0.158		0.211 ±0.147		0.234 ±0.194		0.241 ±0.167		0.245 ±0.168		0.266 ±0.187		0.270 ±0.171		0.273 ±0.168	

HOAs, higher-order aberrations, mean ± SD μm ＊P-value<0.05
