# Supplementary material for: Gender differences in higher-order aberrations and refractive error in Japanese school children: the Kyoto Childhood Refractive Error Study (KRES)
Source: Jpn J Ophthalmol. 2025 Sep 2;70(2):245–53. doi: 10.1007/s10384-025-01272-6 (PMC13091847; doi:10.1007/s10384-025-01272-6)
Supplement: Supplementary file 11 — Supplementary file11 (PDF 168 KB) [file 10384_2025_1272_MOESM11_ESM.pdf]

**Online Resource 11** Questionnaire results

| | **a. Outdoor activity time** ($p=0.06$) | | **c. Television time** ($p=0.35$) | | **d. Mobile-phone-app game time** ($p<0.001$) | |
|---|---|---|---|---|---|---|
| | **Boys** | **Girls** | **Boys** | **Girls** | **Boys** | **Girls** |
| **≧3 hours** | 5.7% | 5.2% | 11.3% | 11.9% | 14.1% | 9.9% |
| **< 3hours** | 10.4% | 9.0% | 19.1% | 20.8% | 14.4% | 9.5% |
| **<2 hours** | 32.5% | 28.2% | 42.4% | 40.8% | 29.2% | 20.2% |
| **<1 hour** | 51.4% | 57.6% | 27.2% | 26.5% | 42.3% | 60.4% |

| | **b. Reading time** ($p<0.001$) | | | **e. Numbers of subjects whose parents wear spectacles** ($p=0.08$) | |
|---|---|---|---|---|---|
| | **Boys** | **Girls** | | **Boys** | **Girls** |
| **≧ 1 hour** | 7.8% | 12.0% | **2 persons** | 26.7% | 24.6% |
| **< 1 hour** | 92.2% | 88.0% | **1 person** | 54.0% | 51.6% |
| | | | **0 persons** | 19.3% | 23.8% |
