# Supplementary material for: Gender differences in higher-order aberrations and refractive error in Japanese school children: the Kyoto Childhood Refractive Error Study (KRES)
Source: Jpn J Ophthalmol. 2025 Sep 2;70(2):245–53. doi: 10.1007/s10384-025-01272-6 (PMC13091847; doi:10.1007/s10384-025-01272-6)
Supplement: Supplementary file 12 — Supplementary file12 (PDF 148 KB) [file 10384_2025_1272_MOESM12_ESM.pdf]

Online Resource 12 Comparison of the mean height and weight between boys and girls (each grade)

		Grade 1 (n=349)	p-value	Grade 2 (n=339)	p-value	Grade 3 (n=355)	p-value	Grade 4 (n=358)	p-value	Grade 5 (n=447)	p-value	Grade 6 (n=499)	p-value	Grade 7 (n=393)	p-value	Grade 8 (n=297)	p-value	Grade 9 (n=224)	p-value
**Height (cm)**	boys	118.2 ±5.6	0.77	124.1 ±5.3	0.99	130.3 ±6.2	0.36	135.0 ±6.9	0.08	140.7 ±7.0	0.08	146.9 ±8.2	0.045 ＊	155.3 ±8.1	0.004 ＊	161.9 ±7.1	<0.001 ＊	166.9 ±6.2	<0.001 ＊
	girls	118.4 ±5.3		124.1 ±5.5		129.7 ±5.9		136.3 ±7.0		141.9 ±7.3		148.3 ±7.0		153.2 ±6.2		156.1 ±6.0		157.7 ±5.9	
**Weight (Kg)**	boys	22.0 ±3.8	0.54	24.6 ±4.5	0.55	28.1 ±5.6	0.54	31.6 ±6.8	0.94	35.9 ±8.7	0.23	40.4 ±9.3	0.99	46.4 ±10.4	0.11	52.0 ±11.6	<0.001 ＊	57.5 ±12.0	<0.001 ＊
	girls	21.7 ±3.6		24.3 ±4.1		27.8 ±5.5		31.7 ±6.6		35.0 ±7.1		40.4 ±8.2		44.8 ±8.2		47.5 ±7.9		49.8 ±8.0	

mean ± SD ＊P-value<0.05
