# Supplementary material for: Gender differences in higher-order aberrations and refractive error in Japanese school children: the Kyoto Childhood Refractive Error Study (KRES)
Source: Jpn J Ophthalmol. 2025 Sep 2;70(2):245–53. doi: 10.1007/s10384-025-01272-6 (PMC13091847; doi:10.1007/s10384-025-01272-6)
Supplement: Supplementary file 13 — Supplementary file13 (PDF 148 KB) [file 10384_2025_1272_MOESM13_ESM.pdf]

Online Resource 13 Results of adjusting height for axial length (n=3261)

		**Estimates**	**95%CI**	***P*-value**
**Axial length**	Gender	-0.518	-0.639, -0.396	<0.001 *
	Height	0.035	0.033, 0.037	<0.001 *

CI, Confidence interval.

Estimated gender differences: reference value is boys, Estimated height differences: reference value is 1cm

* P-value<0.05
