# Supplementary material for: Gender differences in higher-order aberrations and refractive error in Japanese school children: the Kyoto Childhood Refractive Error Study (KRES)
Source: Jpn J Ophthalmol. 2025 Sep 2;70(2):245–53. doi: 10.1007/s10384-025-01272-6 (PMC13091847; doi:10.1007/s10384-025-01272-6)
Supplement: Supplementary file 14 — Supplementary file14 (PDF 258 KB) [file 10384_2025_1272_MOESM14_ESM.pdf]

Online Resource 14 Scatterplot of AL/CR steep and Objective refraction SE ●:Boys ●:Girls

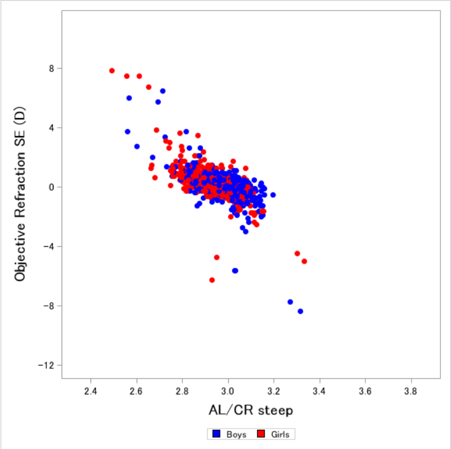

Grade 1

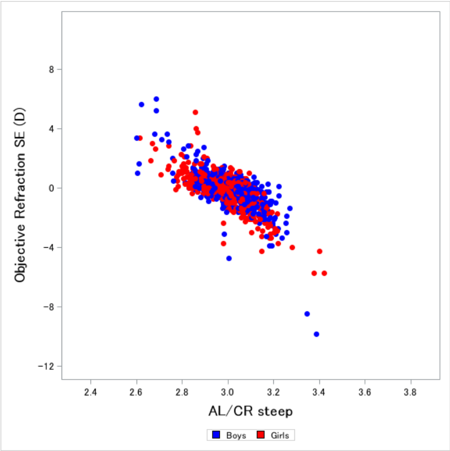

Grade 2

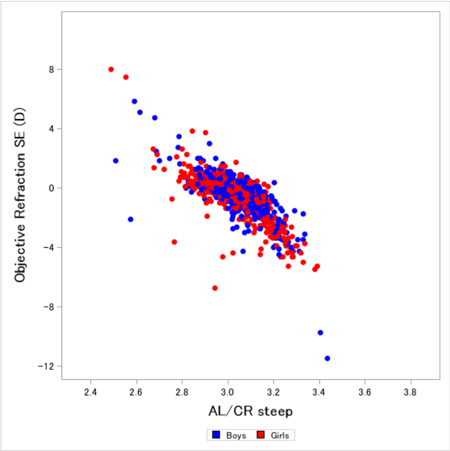

Grade 3

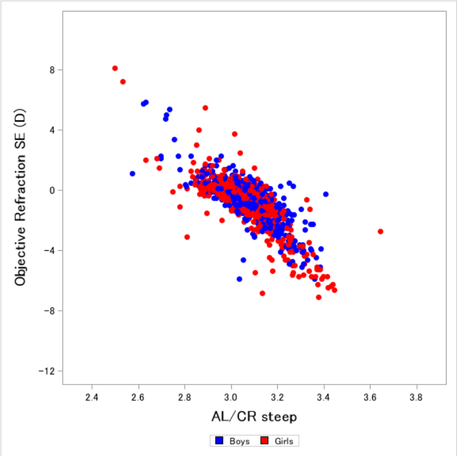

Grade 4

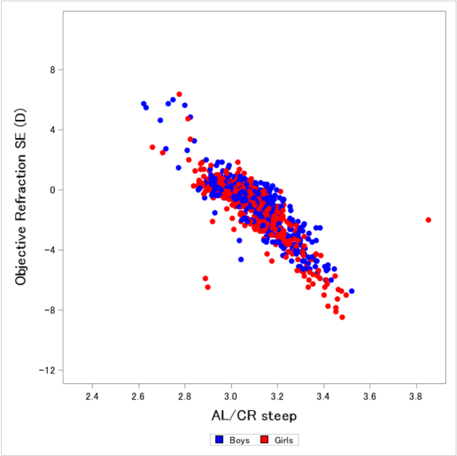

Grade 5

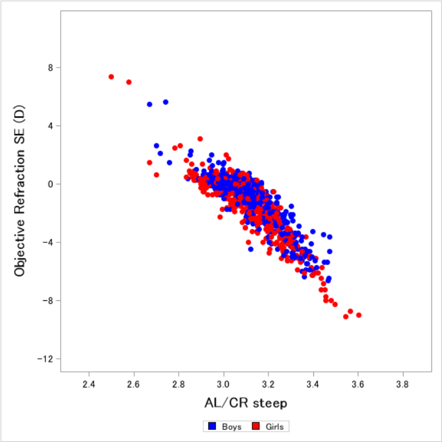

Grade 6

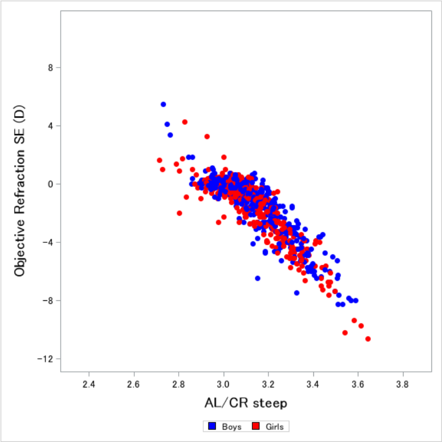

Grade 7

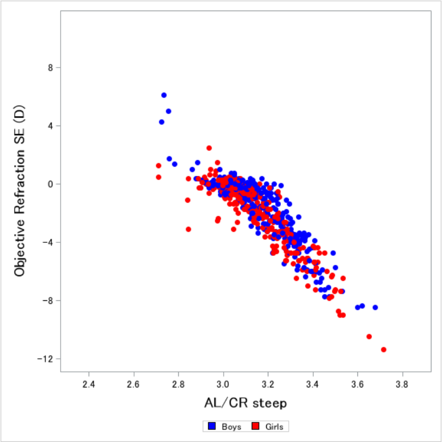

Grade 8

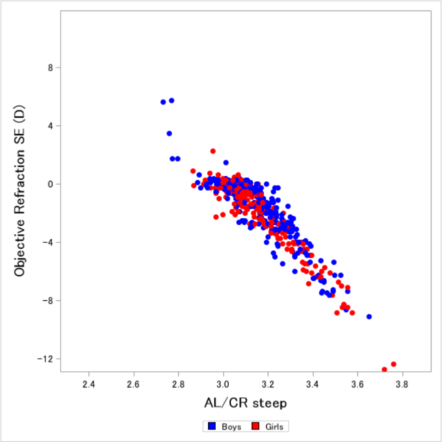

Grade 9
